# Polysaccharides From *Pogostemon cablin* (Blanco) Benth.: Characterization and Antioxidant Activities

**DOI:** 10.3389/fphar.2022.933669

**Published:** 2022-06-16

**Authors:** Lei Zhao, Lei Wang, Zimeng Guo, Ning Zhang, Qisheng Feng, Bo Li

**Affiliations:** ^1^ School of Graduation, Changchun University of Chinese Medicine, Changchun, China; ^2^ School of Pharmaceutical Sciences, Changchun University of Chinese Medicine, Changchun, China

**Keywords:** polysaccharides, *Pogostemon cablin* (Blanco) Benth, chemical characterisation, oxidative stress, monosaccharide composition

## Abstract

Two polysaccharide fractions from *Pogostemon cablin* (Blanco) Benth. (*P. cablin*) (designated as PCB-1 and PCB2-1) were isolated by water extraction and purified by Sepharose chromatography. The chemical properties of the polysaccharides were characterised, and their antioxidant activities were evaluated. The sugar content of the crude polysaccharide (PCB), PCB-1, and PCB2-1 was 58.74, 90.23 and 88.61%, respectively. The molecular weights of PCB-1 and PCB2-1 were determined to be 97.8 and 12.8 kDa, respectively. Monosaccharide composition analysis showed that all the three polysaccharides consisted of mannose, rhamnose, galacturonic acid, galactose, glucose, and arabinose, but with varying molar ratios. The polysaccharides exhibited significantly high antioxidant activities *in vitro* based on the scavenging activity against hydroxyl radicals, metal ion-chelating and ferric-reducing abilities. *In vivo* experiments in an oxidatively damaged mice model showed that PCB-1 increased the levels of antioxidant enzymes, including superoxide dismutase, catalase, and glutathione peroxidase, and inhibited malondialdehyde formation in the serum and liver. These findings suggest that PCB-1 has significant potential as an antioxidant in functional foods.

## Introduction

Antioxidant activity is necessary for the body to defend against and resist the progression of various diseases, including aging linked to excessive reactive oxygen species (ROS) production (Huang et al., 2021). An excessive amounts of ROS can be produced by environmental stimuli to cause oxidative damage to DNA, protein, and lipids (Ke et al., 2009). Under normal conditions, the antioxidant defence systems can quickly remove excess ROS; however, these protective systems may not be effective under pathological conditions. Thus, the importance of identifying and understanding the role of dietary antioxidants in defending against cumulative oxidative stress and the underlying mechanism is increasingly being recognised.

Polysaccharides, as essential functional components of most plants, typically exhibit highly specific chemical structures and biological activities, such as immunity-enhancing, anti-aging, blood sugar-reducing, blood lipid-reducing, anti-tumour, anti-viral, anti-bacterial, and anti-coagulation effects (Li. et al., 2021b; Chumroenphat, 2021). Moreover, previous studies have demonstrated that polysaccharides have low toxicities and no side effects when used for a disease treatment (Basak and Gokhale, 2022). Most polysaccharides can relieve oxidative stress damage (Liu et al., 2021; Wang et al., 2021). In particular, polysaccharides have been shown to influence oxidative damage in mice via their antioxidant activities by acting on sirtuin 1 (SIRT1), a key member of the family of silent transcriptional regulators. SIRT1 can deacetylate a variety of proteins and plays an important role in resisting oxidative damage. Moreover, it is also an upstream regulator of peroxisome proliferator-activated receptor-gamma coactivator-1α (PGC-1α) (Lee et al., 2018; Waldman et al., 2018). SIRT1 can activate the expression of PGC-1α through deacetylation, thereby reducing the damage by oxidative stress. The uncoupling protein 2 (UCP2) is an inner-membrane mitochondrial protein, which is an important regulator of ROS formation, and PGC-1α can directly regulate the expression of UCP2 (Huang et al., 2019).

“Guang-Huo-Xiang” is a traditional medicine derived from the dry overground parts of *Pogostemon cablin* (Blanco) Benth (*P. cablin*), which is mainly distributed in China, India, and Indonesia (Su et al., 2017). *P. cablin* is well-known for its oil, which is used as food additive or in the perfume and cosmetic industries (Swamy and Sinniah, 2015). *P. cablin* has been included on the list of medicinal and food homology in China (Kim et al., 2015), but has recently attracted more attention because of its varied biological functions, including antibacterial (Wan et al., 2021), antiviral (Yu et al., 2019), anti-inflammatory (Chen et al., 2021), antidepressant (Zhuo et al., 2020), and anti-oxidative effects (Liu et al., 2017). However, previous investigations on *P. cablin* have mainly focused on the constituent mono- and sesquiterpenoids, triterpenoids, steroids, flavonoids, alkaloids, and phenylpropanoid glycosides (Zhao et al., 2005). To date, no specific studies on the structural characterisation and biological activities of *P. cablin* polysaccharides have been conducted, specifically in terms of their antioxidant activity. Therefore, our study on the antioxidant activity of *P. cablin* polysaccharides is of great significance for the development and utilization of *P. cablin*.

In the current study, we purified the polysaccharide fractions from *P. cablin* and the antioxidant activity of these polysaccharides was measured *in vitro*. In addition, the effect of one of the extracted polysaccharides on antioxidant enzymes was evaluated *in vivo* using the mice model with stimulated oxidative damage. Therefore, this study aimed to extract *P. cablin* polysaccharide and fractionate it systematically by using gel chromatography, and to further explore their antioxidation potential *in vitro* and *in vivo.*

## Materials and Methods

### Extraction and Purification of Polysaccharides

**FIGURE 1:**
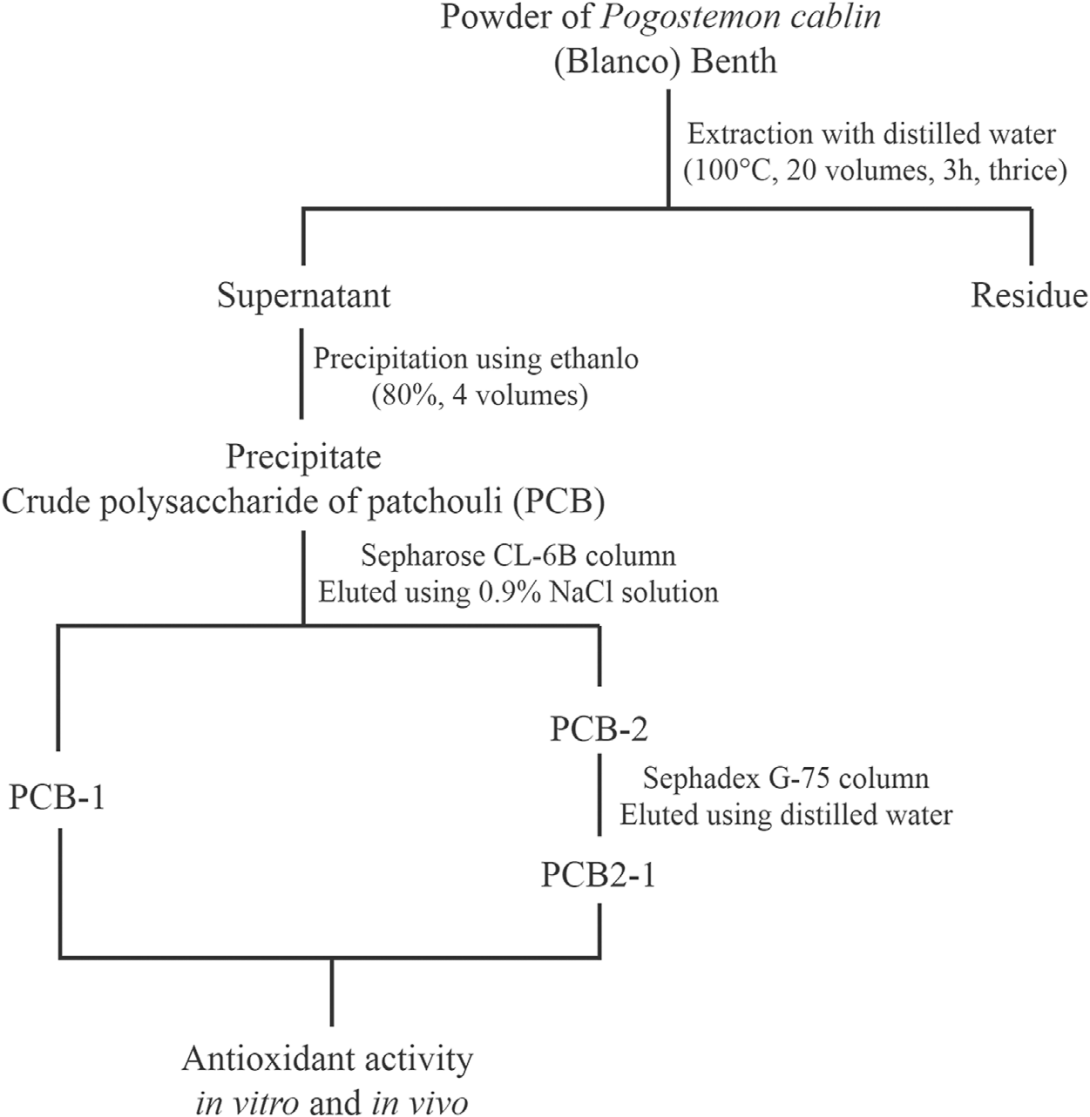
Experimental procedure for extraction and purification of polysaccharides isolated from *P. cablin*.

The *P. cablin* powder was obtained from a local shop (Changchun, Jilin Province, China), and its taxonomical characteristics were identified by Professor Shumin Wang. Samples were extracted at 100°C in a water bath at a ratio of 1: 20 (w/v) three times for 3, 2, and 2 h. The supernatants were added to four volumes of 80% ethanol for 24 h and the retentates were dissolved in distilled water. The proteins were then removed using Sevage solution (chloroform: n-butyl alcohol, 4:1, v/v) (Zhu et al., 2019). The dialysis liquid was collected and lyophilised to obtain the crude polysaccharide (PCB). PCB was dissolved in deionised water at a concentration of 1 mg/ml and centrifuged (11,000 × g, 10 min). The supernatants were loaded onto a Sepharose CL-6B chromatography column (2.5 × 90 cm) and eluted with NaCl solution (0.9%) at a flow rate of 0.5 ml/min. Two fractions were obtained (designated as PCB-1 and PCB-2). A Sephadex G-75 column with a column size of 2.5 × 90 cm was applied to further purify PCB-2, which was eluted with distilled water at a flow rate of 0.5 ml/min. The eluting peak was collected (designated as PCB2-1). The experimental procedure is shown in Figure 1.

### Molecular Weight Determination

The average Mw of PCB-1 and PCB2-1 were evaluated using high-performance gel permeation chromatography (HPGPC) (Xu et al., 2016). The samples (10 mg) were dissolved with ultrapure water (1.0 ml) and filtered through a 0.45 μm membrane filter. Analysis was performed with an Ultimate 3,000 system (Thermo Fisher Scientific, United States) coupled to a TSK-G3000 PW_XL_ column (7.8 mm i. d. × 30.0 cm) and tested by a refractive index detector (RID-10A) at 40°C. The column was eluted with ultrapure water at a flow rate of 0.5 ml/min, and the filtrate solution (20 μL) was injected for high performance liquid chromatography (HPLC) analysis.

### Chemical Analysis

The polysaccharide content was measured by the phenol-sulfuric acid method with glucose as a standard (Du et al., 2016). Uronic acid was determined using the previously reported method with galacturonic acid as the standard (Blumenkrantz and Asboe-Hansen, 1973). Protein content was measured by Bradford assay at 595 nm using bovine serum albumin as a standard (Sedmak and Grossberg, 1977).

### Monosaccharide Composition Analysis

The monosaccharide composition was analysed using the previous method with some modifications (Li et al., 2018). Briefly, the samples (2.0 mg) were hydrolysed in 0.5 ml of 1 M hydrochloric acid (dissolved in methanol) at 80°C for 16 h, then further hydrolysed with 0.5 ml of 2 M trifluoroacetic acid at 120°C for 1 h. After the excess acid was removed by evaporation, the dried product was mixed with aqueous sodium hydroxide (0.3 M, 0.5 ml). The mixtures were derived with 0.5 ml of 1-phenyl-3-methyl-5-pyrazolone (PMP) and 0.5 ml of 0.3 M sodium hydroxide. The obtained product was neutralised with 50 μL of hydrochloric acid (0.3 M), and excess PMP reagents were removed using 1 ml of chloroform, repeated thrice.

The PMP derivatives (20 μL) were analysed using the Agilent RRLC 1200 SL system (Agilent Technologies, DE, Wilmington, United States), coupled with a DIKMA Inertsil ODS-3 column (4.6 × 150 mm, 5 μm, Dikma, Japan). The derivatives were eluted with the mobile phase, composed of 82.0% phosphate-buffered saline (0.1 M, pH 7.0) and 18.0% acetonitrile (v/v). Total HPLC run at a flow rate of 1.0 ml/min, and the absorbance was measured at 245 nm.

### Fourier-Transform Infrared Spectroscopy Analysis

FT-IR spectroscopy of the samples was performed according to a previous study (Jiao et al., 2020). The IR spectra were acquired using an FT-IR spectrometer at 25°C. Briefly, the sample (1.5 mg) was measured with potassium bromide (150 mg) powder pellets on a Bruker Vertex 7.0 FT-IR spectrometer (Germany). The scan range was 4,000–400 cm^−1^.

### *In Vitro* Antioxidant Activities

#### Hydroxyl Radical Scavenging Activities

The hydroxyl radical (•OH)-scavenging activities of PCB-1 and PCB2-1 were measured following the method described by Jiao et al. (Jiao et al., 2014). Briefly, 0.1 ml sample solution was added to 0.6 ml of a reaction mixture [phosphate buffer (0.2 M, pH = 7.4), deoxyribose (2.67 mM), and ethylene diamine tetraacetie acid (EDTA, 0.13 mM)], FeSO_4_ (0.2 ml, 0.4 mM), vitamin C (Vc, 0.05 ml, 12 mM), and H_2_O_2_ (0.05 ml, 20 mM). The working mixtures were incubated together (37°C, 15 min). Then, thiobarbituric acid (TBA, 1 ml, 1%) and trichloroacetic acid (1 ml, 1%) were mixed evenly and incubated together at 100°C for 15 min. The absorbance of each mixture was measured at 532 nm using a UV spectrophotometer. Distilled water was used as a control and Vc as positive control for the determination of •OH. Radical scavenging activity was calculated using the following equation:

$$\mathrm{Scavenging}\;\mathrm{rate}\left(\%\right)=\left[A_{0}-\left(A_{s}-A_{i}\right)\right]/A_{0}\times 100$$

where A_s_ is the absorbance of the sample, A_0_ is the absorbance of the control group, and A_i_ is the absorbance of the mixture without FeSO_4_.

### Chelating Ability on Ferrous Ions

The chelating ability on ferrous ions was determined according to a previous study (Ge et al., 2014). The polysaccharide sample (1 ml) was mixed with methanol (3.7 ml) solution and FeCl_2_·4H_2_O (2 mM, 0.1 ml), and then ferrozine (5 mM, 0.2 ml) was added to the mixture and shaken well.

After incubation at room temperature for 10 min, the absorbance was measured at 562 nm against a control. Distilled water as the control and EDTA was used as positive control for the determination of chelating ability on ferrous ions. The scavenging of ferrous ions was calculated using the following equation:

$$\mathrm{Scavenging}\;\mathrm{rate}\left(\%\right)=\left(A_{0}-A_{s}\right)/A_{0}\times 100$$

where A_0_ is the absorbance of the control group and A_s_ is the absorbance of the sample.

#### Ferric-Reducing Antioxidant Power

The FRAP abilities of samples were determined according to the minor method of Dammak et al. (2018) with slight modifications. Firstly, various concentrations of sample (0.3 ml) was reacted with 2.7 ml of freshly prepared FRAP reagent [5.0 ml of 10 mM 1,3,5- tri (2-pyridyl)-2,4,6-triazine (TPTZ) in HCl (40 mM), 5.0 ml of 20 mM FeCl_3_·6H_2_O, and 50 ml of 300 mM acetate buffer, pH 6.3]. After being shaken well and incubated together at 37°C for 10 min, the absorbance of the resulting mixture was measured at 593 nm. A higher FRAP value indicates stronger antioxidant capacity. Distilled water was used as the control and EDTA was used as the positive control for the determination of FRAP.

### Determination of *in Vivo* Antioxidant Capacity

#### Animals, Grouping, and Experimental Design

The antioxidant activities of PCB-1 *in vivo* were determined according to a previously reported method (Zeng et al., 2018). Male ICR mice (8 weeks old, 18–22 g; Yisi Experimental Animal Technology Co., Ltd., Changchun, Jilin Province, China) were maintained on a 12-h-dark/12-h-light cycle at approximately 22°C and 50–60% relative humidity with free access to food and water. All animal studies have been approved by the Animal Ethics Committee of Changchun University of Chinese Medicine.

After adaptation to their environment for 1 week, 60 mice were randomly divided into six groups (*n* = 10 per group): normal control group (NCG), d-galactose (D-gal) model control group (MCG), Vc positive control group (PCG), and dose-dependent PCB-1 (50, 100, and 200 mg/kg body weight) treatment groups. With the exception of the NCG group, the mice were subcutaneously injected with 1.35 g/kg body weight D-gal. The PCG group was orally administered 100 mg/kg body weight Vc; the three treatment groups were orally administered 50, 100, and 200 mg/kg body weight PCB-1, respectively; and the NCG and MCG groups were orally administered an equal dose of normal saline (Ye et al., 2014). Subcutaneous injection and oral administration were calculated as 0.1 ml/10 g. All mice were treated for 42 consecutive days, once daily.

#### Biochemical Assay

The mice were weighed and killed by decapitation the following morning of the last drug administration. Blood samples were collected and centrifuged (4,000 × *g*, 10 min, 4°C) and the serum was collected. The liver was removed, weighed, and immediately stored in 0.1 g tissue/mL ice-cold isotonic physiological saline (Qi et al., 2020). The samples were centrifuged as described above, and the supernatant was collected and subjected to further analysis.

The commercial reagent kits obtained from Nanjing Jiancheng Bioengineering Institute (Jiangsu, China) were used to analyse the activities of superoxide dismutase (SOD), catalase (CAT), and glutathione peroxidase (GSH-Px), and to determine malondialdehyde (MDA) levels and the protein content. SOD, CAT, and GSH-Px activities were determined using xanthine oxidase-xanthine reaction system, yellow H_2_O_2_-ammonium molybdate reaction system, and reduced glutathione (GSH)- H_2_O_2_ reaction system methods, respectively. The levels of MDA were measured using the TBA method and the ferric reducing/antioxidant power reaction system.

#### Western Blot Analysis

Frozen mice liver (40 mg) were thawed, minced, and homogenised on ice using an Ultraturrax homogeniser in RIPA lysis buffer (500 μL). The RIPA tissue lysate (Thermo Fisher Scientific, Inc., Waltham, MA, United States) was used to extract total protein from the liver tissues. Homogenates were centrifuged (10,000 × *g*, 20 min, 4°C) to obtain the supernatants. The protein concentrations were analysed using BCA protein quantification kit (Nanjing Jiancheng Bioengineering Institute, Jiangsu, China). Protein lysates (40 μg) were separated by electrophoresis and then transferred to a polyvinylidene fluoride (PVDF) membrane (Millipore, Billerica, MA, United States) overnight at 4°C. The PVDF membrane was then blocked with skimmed milk (5%) for 1 h at 25°C and shaken at 75 rpm, followed by incubation with primary antibodies anti-β-actin (1:5,000, Abcam, United Kingdom), anti-SIRT1 (1:3,000, ABclonal, United States), anti-PGC-1α (1:2,000, Bioss, United States), and anti-UCP2 (1:1,500, Bioss, United States) at 4°C overnight and then with a secondary antibody (1:3,000, Servicebio, Wuhan, China) for 1 h at 25°C. The signals were visualised by chemiluminescence using enhanced chemiluminescence reagents and X-ray films. The strip images were scanned and the optical densities of the protein bands were quantified using ImageJ software (Zhou et al., 2021).

### Data Analysis

All data are expressed as the mean ± standard deviation. The statistical significance of the difference between groups was evaluated using one-way analysis of variance followed by Student’s *t*-test. Significance level was set at *p* < 0.05.

## Results and Discussion

### Purification and Characterisation of Polysaccharides

**FIGURE 2:**
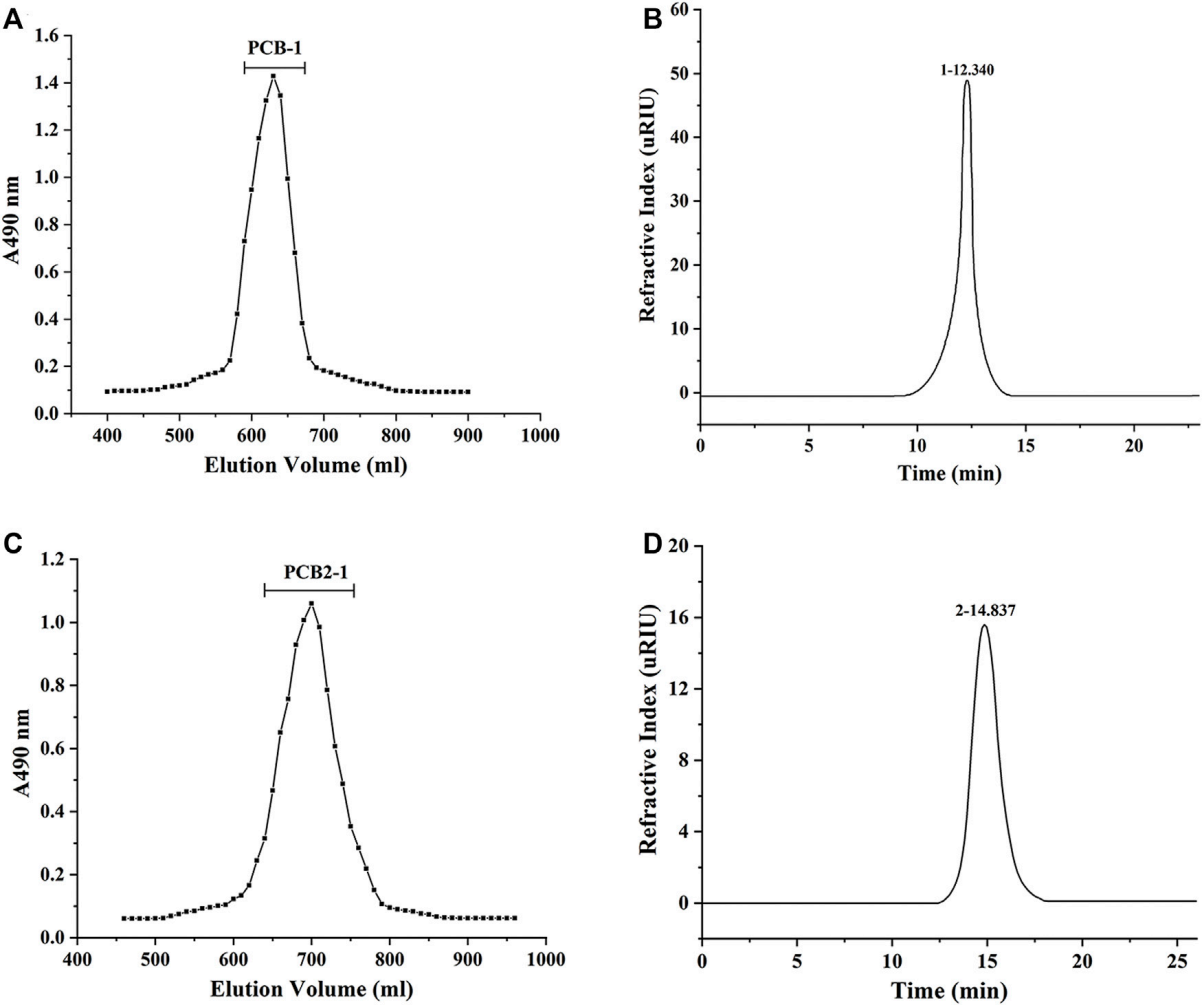
Molecular weight (Mw) determination of polysaccharides isolated from *P. cablin*: **(A)** HPGPC analysis of PCB-1; **(B)** HPLC analysis of PCB-1; **(C)** HPGPC analysis of PCB2-1; **(D)** HPLC analysis of PCB2-1.

Using hot water extraction, alcohol precipitation, deproteinisation, and dialysis, PCB was obtained from *P. cablin*, with a yield of 6.24%. The PCB was purified using a Sepharose CL-6B column, and two fractions were obtained, designated as PCB-1 (yield of 3.26%) and PCB-2 (yield of 2.23%). PCB-2 was further fractionated by Sephadex G-75 gel permeation chromatography and the target polysaccharide obtained was designated as PCB2-1 (yield of 1.96%). The elution curves of PCB-1 and PCB2-1 are shown in Figures 2A,C. The HPLC profiles of both PCB-1 and PCB2-1 had a single and symmetrical sharp peak, indicating that they were homogeneous polysaccharides (Figures 2B,D). The Mw of PCB-1 was determined to be 97.8 kDa, whereas that of PCB2-1 was 12.8 kDa. The carbohydrate contents of PCB, PCB-1, and PCB2-1 was 58.74, 90.23, and 88.61%, and the uronic acid content was 7.65, 13.04, and 9.10%, respectively. Moreover, a negative response to the Bradford reaction, proved that the protein had been effectively removed from the samples (Zeng et al., 2019).

**FIGURE 3:**
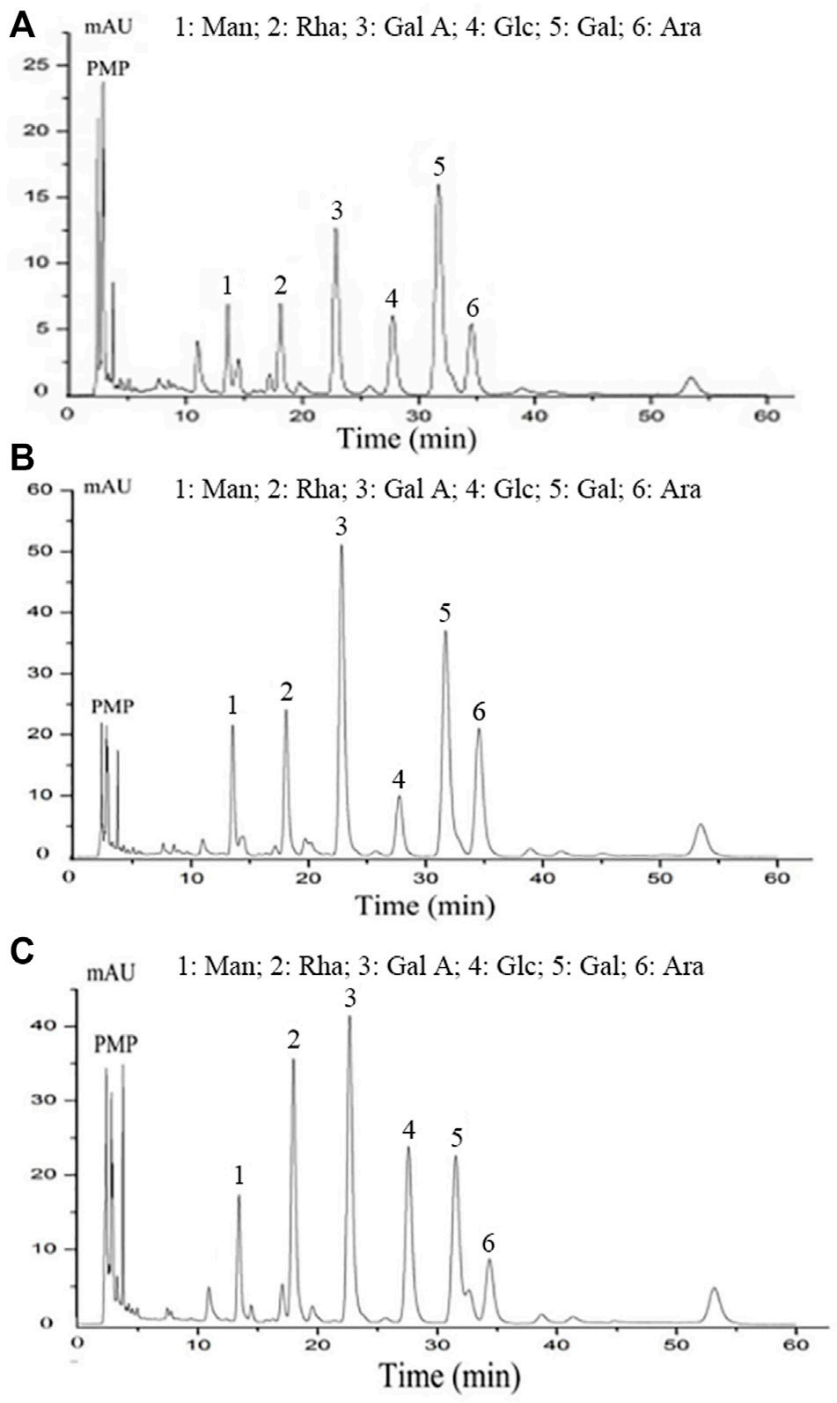
Monosaccharide composition analysis of polysaccharides isolated from *P. cablin*: **(A)** Monosaccharide composition of PCB; **(B)** Monosaccharide composition of PCB-1; **(C)** Monosaccharide composition of PCB2-1. PMP-labeled and analysed by HPLC.

The monosaccharide composition is of great significance for the characterisation of polysaccharides given the important effects of monosaccharides on structure and biological activity (Chen et al., 2019). HPLC analysis showed that PCB (Figure 3A), PCB-1 (Figure 3B), and PCB2-1 (Figure 3C) were acidic heteropolysaccharides. Although all these polysaccharides were mainly composed of mannose (Man), galactose (Gal), galacturonic acid (Gal A), rhamnose (Rha), glucose (Glc) and arabinose (Ara); however, there were differences in the content of specific monosaccharides and their ratios with molar ratios of 1.00:5.17:2.66:1.39:1.56:2.39, 1.00:3.63:4.31:1.73:0.97:2.93, and 1.00:2.51:3.48:3.20:2.70:1.22 for PCB, PCB-1, and PCB2-1, respectively. Among PCB-1 and PCB2-1, the content of Gal A was the highest, which was 29.58 and 24.66%, respectively. Moreover, Chen et al. obtained two polysaccharides from *P. cablin* by combination of water extraction and ion exchange chromatography. Of note, Gal and Glc were the major component in the two polysaccharides, and the content of Gal A in the two polysaccharide was less than 10%, which was different from this study (Chen et al., 2020b). The difference in the composition of monosaccharides might be attributed to the extraction and purification procedures (Li et al., 2019; Li et al., 2021).

### FT-IR Spectrum Analysis

**FIGURE 4:**
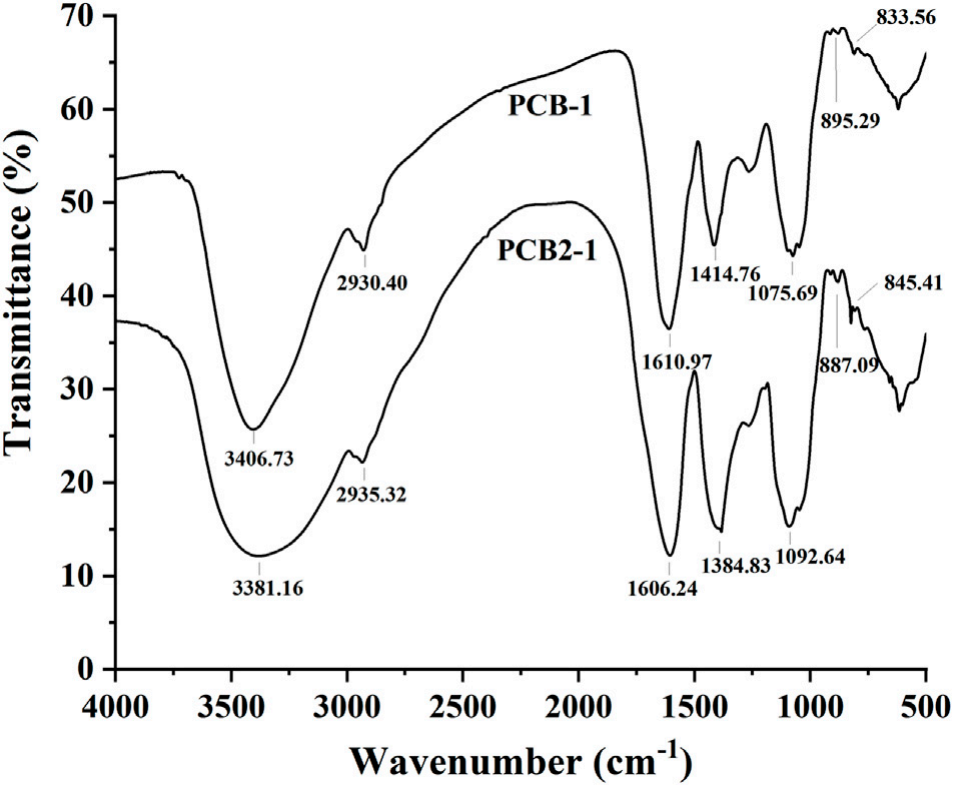
FT-IR spectra of polysaccharides isolated from *P. cablin*: FT-IR spectrum of PCB-1 and PCB2-1.

As shown in Figure 4, the absorption bands in the range of 4000–400 cm^−1^ were recognised as characteristic peaks of polysaccharides (Hashemifesharaki et al., 2020). The peaks at 3406.73 and 3381.16 cm^−1^ were typical peaks belonging to O–H bond stretching vibrations present in PCB-1 and PCB2-1, and the absorption bands at 2930.40 and 2935.32 cm^−1^ were attributed to C–H stretching vibrations in PCB-1 and PCB2-1, including CH_2_ and CH_3_ groups. In addition, the peaks at 1414.76 and 1384.83 cm^−1^ attributed to the stretching vibration signals of C–H and C–O were absent in PCB-1 and PCB2-1. Moreover, the strong characteristic absorption bands appearing at 895.29, and 887.09 cm^−1^ indicated the abundance of β-glycosidic linkages in PCB-1 and PCB2-1, and the signals at 833.56 and 845.41 cm^−1^ suggested the existence of an α-terminal epimer in these polysaccharides (Hua et al., 2019). Thus, the glycosidic bonds in these *P. cablin* polysaccharides could be identified as α-type and β-type (Chen et al., 2019b).

### *In Vitro* Antioxidant Activities

#### Hydroxyl Radical Scavenging Activity

The •OH are considered highly reactive oxygen radicals, which can stimulate the peroxidation reaction of nucleic acids, protein, and lipids (Chen et al., 2020a). Hence, removal of •OH is crucial for effective antioxidant activity to protect cells from damage (Qi et al., 2006). As shown in the scavenging activities of the samples in a dose-dependent manner increased in the concentration range of 0.25–7.5 mg/ml in the order Vc > PCB-1 > PCB2-1. Moreover, PCB-1 exhibited the strongest antioxidant activity at a concentration of 7.5 mg/ml, with a scavenging rate of 81.29%, whereas PCB2-1 had lower scavenging activity at the same concentration (40.25%). Therefore, PCB-1 exhibits strong capacity to supply hydrogen to combine with •OH, thereby achieving a radical scavenging effect.

It has been widely reported that the antioxidant activity of polysaccharides to scavenge •OH depends on the type, number, and position of anomeric hydrogen, monosaccharide components, and chemical structures (Chumroenphat, 2021). Thus, the stronger ability of PCB-1 to inhibit the •OH may be related to its higher Mw compared to that of PCB2-1. However, the in-depth structure–activity relationship requires further study.

#### Metal Chelating Ability

**FIGURE 5:**
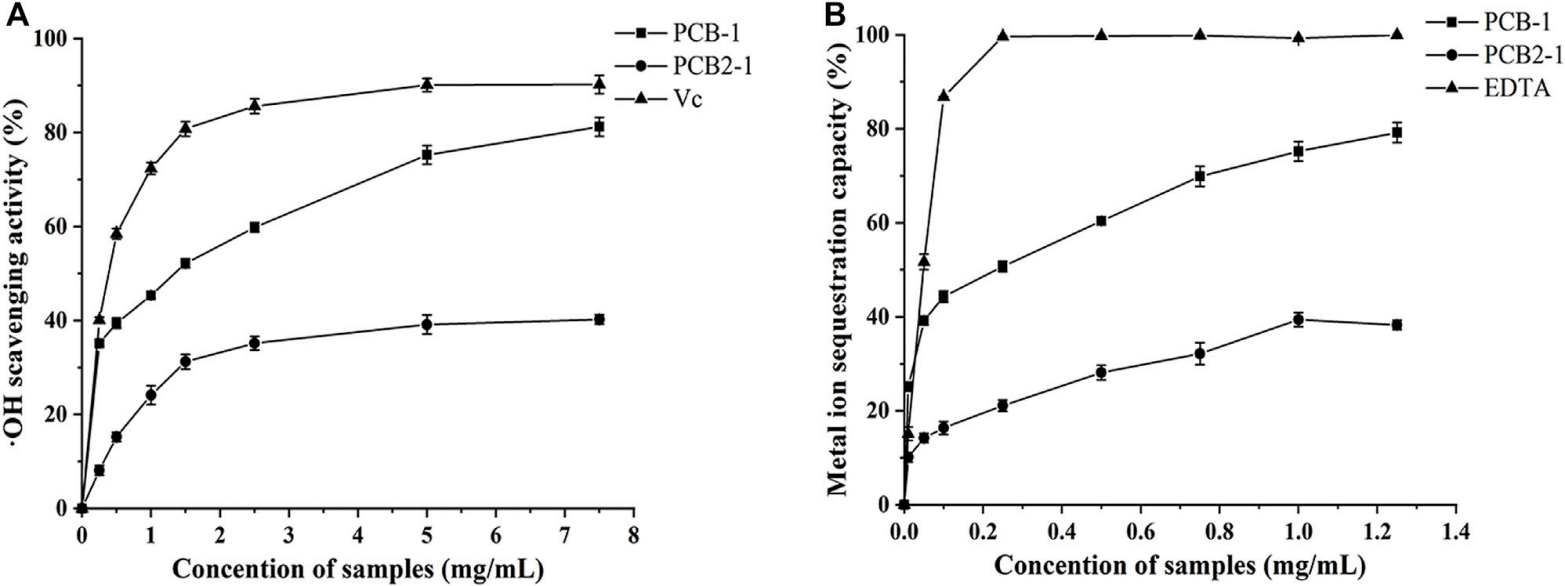
Antioxidant activities of the polysaccharides from *P. cablin*: **(A)** Hydroxyl radical scavenging activity of PCB-1 and PCB2-1 **(B)** Fe^2+^ chelating activity of PCB-1 and PCB2-1. Each value represents the mean ± SD (*n* = 3).

Iron can stimulate lipid peroxidation through the Fenton reaction (Fe^2+^ + H_2_O_2_ → Fe^3+^ + OH^•^ + OH^−^), which accelerates lipid peroxidation by breaking down hydrogen and driving the chain reaction of lipid peroxidation (Sun et al., 2010). Thus, Fe^2+^ plays an important role in antioxidation and is a significant co-oxidant in cells. The chelating assay involves inhibition of the formation of red-coloured ferrozine-Fe^2+^ complex to indicate antioxidant activity (Liu et al., 2010). The chelating abilities of PCB-1, PCB2-1, and EDTA are shown in Figure 5B. PCB-1 achieved maximum chelating ability of 79.2% at 1.25 mg/ml, which was higher than that of PCB2-1 at the same concentration. EDTA and both polysaccharides displayed significant antioxidant capacity on Fe^2+^ in a concentration-dependent manner. Yuan et al. found that compounds with better metal chelating capacities always include two or more of the following functional groups: -OH, -SH, -COOH, -PO_3_H_2_, C=O, -NR_2_, -S-, and -O- (Yuan et al., 2005). Therefore, these results reveal a marked capacity of PCB-1 and PCB2-1 for iron binding, suggesting that the chelating activities may be due to the uronic acid content.

#### Ferric Reducing Ability

As a significant indicator of antioxidant activity, the FRAP assay is commonly used to determine the antioxidant activity of polysaccharides, as it is a simple, rapid, and sensitive test (Liu et al., 2021). The antioxidant activity was evaluated by detecting the increase in absorbance that leads to formation of the Fe^3+^–TPTZ complex, which was detected by the change in absorbance at 593 nm (Veenashri and Muralikrishna, 2011). At a concentration of 1.25 mg/ml, the FRAP value of PCB-1 and PCB2-1 was 4.52 and 1.16 mmol FeSO_4_/g, respectively, suggesting that PCB-1 exhibited higher reducing power. This difference may be related to the differences in the main electron-donating sugar units, the type and position of glycosidic linkages, conformations, and degree of branching of the two polysaccharides (Chumroenphat, 2021).

### *In Vivo* Antioxidation Effects

D-gal was used to establish an oxidative damage mouse model to determine the *in vivo* antioxidant activity of PCB-1. D-gal can prompt the accumulation of ROS, or indirectly decrease free radical production by the formation of advanced glycation end-products *in vivo* (Song et al., 1999; Hsieh et al., 2009). SOD, CAT, and GSH-Px, which are regarded as the major antioxidant enzymes, were used as biomarkers to indicate ROS production and inhibition of the formation of ROS during oxidative stress (Inal et al., 2001). When compared with those of the NCG group, significant decreases in SOD, GSH-Px, and CAT activities were observed in the MCG group, whereas the levels of MDA significantly increased in the serum and liver of MCG mice. The effects of PCB-1 and Vc on the activities of SOD, GSH-Px, CAT, and MDA levels in the serum and livers of oxidatively damaged mice are presented in Table 1 and Table 2. In both the serum and liver, treatment of PCB-1 at 100 or 200 mg/kg body weight and Vc significantly increased the capacities of antioxidant enzymes as compared to those of the MCG group. MDA is the main marker of endogenous lipid peroxidation. Thus, MDA levels can represent the degree of lipid peroxidation (Bagchi et al., 1995). The levels of MDA in the PCB-1 and Vc treatment groups decreased notably in both the serum and liver compared with those of the MCG group. These results further confirm that the inhibitory effect of PCB-1 on antioxidant activities might be, at least in part, due to enhancement in the activities of SOD, CAT, and GSH-Px and a decrease in MDA levels.

**TABLE 1:** Effects of PCB-1 on activities of SOD, CAT, GSH-Px and levels of MDA in serum of D-gal induces oxidative damage mice.

Group	SOD (U/ml)	CAT (U/ml)	GSH-Px (U/ml)	MDA (nmol/ml)
Normal control group	527.11 ± 13.24*	222.84 ± 15.14*	721.14 ± 25.74*	16.89 ± 0.71*
Model control group	360.02 ± 9.74^#^	136.72 ± 10.02^#^	409.87 ± 15.92^#^	36.10 ± 1.02^#^
Positive control group	506.29 ± 18.74*	201.49 ± 11.24**	699.72 ± 21.67**	20.17 ± 1.89*
PCB-1 (50 mg/kg)	370.22 ± 11.87*	140.02 ± 8.79*	467.84 ± 32.66**	35.17 ± 1.19*
PCB-1 (100 mg/kg)	437.65 ± 21.14*	180.07 ± 9.11*	593.69 ± 29.11*	27.29 ± 2.01*
PCB-1 (200 mg/kg)	498.95 ± 22.06	204.79 ± 7.55**	714.01 ± 49.76*	19.72 ± 1.04*

All values were expressed as mean ± SD (*n* = 10 per group). ^#^ *p* < 0.05, ^##^ *p* < 0.01 compared with normal control group; *p < 0.05, **p < 0.01 compared with model control group.

**TABLE 2:** Effects of PCB-1 on activities of SOD, CAT, GSH-Px and levels of MDA in liver of D-gal induces oxidative damage mice.

Group	SOD (U/mg pro)	CAT (U/mg pro)	GSH-Px (U/mg pro)	MDA (nmol/mg pro)
Normal control group	212.09 ± 8.01*	42.39 ± 3.71*	1789.11 ± 87.92*	2.12 ± 0.19*
Model control group	130.77 ± 10.92^#^	25.09 ± 4.87^#^	908.45 ± 35.11^#^	3.94 ± 0.19^#^
Positive control group	198.35 ± 18.42**	39.75 ± 2.17*	1697.00 ± 42.04	2.48 ± 0.18**
PCB-1 (50 mg/kg)	155.67 ± 12.78**	29.92 ± 1.78*	1074.27 ± 59.87**	3.58 ± 0.17**
PCB-1 (100 mg/kg)	178.09 ± 7.54**	32.01 ± 1.24*	1501.32 ± 39.11*	3.09 ± 0.21**
PCB-1 (200 mg/kg)	201.25 ± 13.86**	38.69 ± 1.94*	1717.87 ± 42.48*	2.37 ± 0.20**

All values were expressed as mean ± SD (*n* = 10 per group). ^#^ *p* < 0.05, ^##^ *p* < 0.01 compared with normal control group; *p < 0.05, **p < 0.01 compared with model control group.

### Western Blot Assay

**FIGURE 6:**
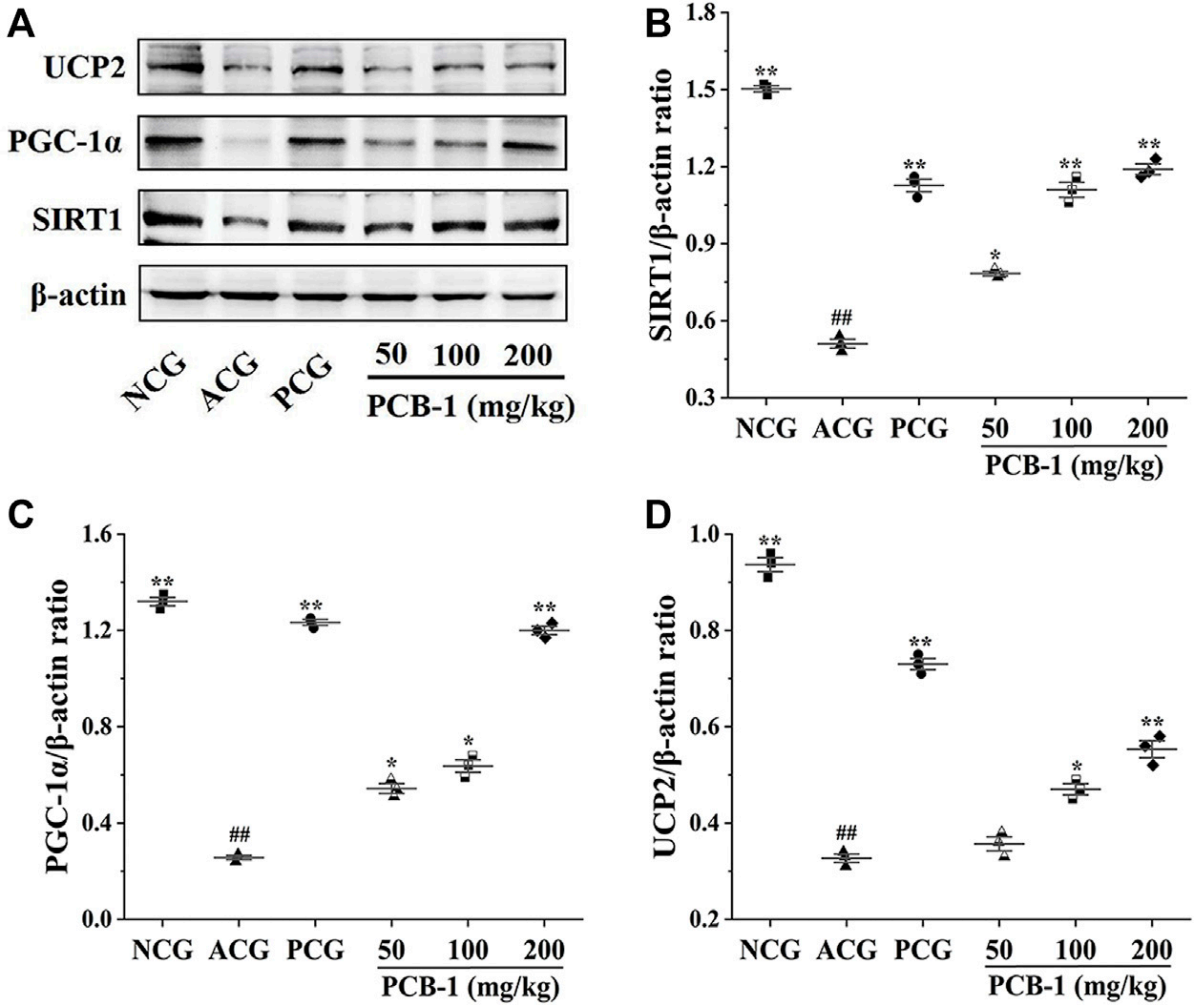
Representative picture of western blot bands and quantification of data showing the effects of PCB-1 alleviated oxidative stress in the liver of mice: **(A)** UCP2, PGC-1α and SIRT1 expression in all experimental groups as evaluated by Western blotting; **(B–D)** Respective histograms of UCP2, PGC-1α and SIRT1 protein expression using Image software. The gray value of the bands was normalised to β-actin (*n* = 3). All bar graph data are presented as mean ± SD. ^#^ *p* < 0.05, ^##^ *p* < 0.01 compared with normal control group; *p < 0.05, **p < 0.01 compared with model control group.

SIRT1 is a deacetylase that regulates processes such as oxidative stress, apoptosis, and neuronal protection (Haigis and Sinclair, 2010). SIRT1-mediated protein deacetylation subsequently activates downstream targets, including PGC-1α and FOXO1 (Huang et al., 2019). PGC-1α has been reported to inhibit apoptosis, reduce ROS accumulation, and protect cells from oxidative stress by activating transcription factors (Ye et al., 2014). Accumulating evidence further implicates UCP2 in protecting against oxidative stress (Anedda et al., 2008). As shown in Figure 6, the MCG group showed a substantial decrease of SIRT1, PGC-1α, and UCP2 expression levels in the liver compared with those of the NCG group (*p* < 0.01), indicating that activation of these proteins could be potentially protective against oxidative stress. Treatment of oxidatively damaged mice with Vc and PCB-1 resulted in a significant increase in SIRT1, PGC-1α, and UCP2 expression levels compared with those of the MCG group, indicating that PCB-1 could help to resist oxidative stress damage (*p* < 0.01). These results further support the beneficial roles of SIRT1, PGC-1α, and UCP2 in the regulation of oxidative stress. In conclusion, the results suggest that the antioxidant effects of PCB-1 could be mediated via the SIRT1/PGC-1α/UCP2 signalling pathway.

## Conclusion

In this study, the polysaccharides were extracted by water extraction and ethanol precipitation from *P. cablin*, and two polysaccharides were obtained from the crude polysaccharide (PCB): PCB-1 (97.8 kDa) and PCB2-1 (12.8 kDa). The yields of PCB, PCB-1, and PCB2-1 were 6.24, 3.26, and 1.96%, respectively. Monosaccharide composition analysis of six monosaccharides (Man, Rha, GalA, Glc, Gal, and Ara) in PCB-1 and PCB2-1, with higher contents of acidic monosaccharides (GalA, Gal, and Ara), indicated that *P. cablin* polysaccharides are mainly acidic sugars. FT-IR spectrum analysis showed that *P. cablin* polysaccharides contained both α- and β-configuration sugars. PCB-1 had better antioxidant activity than PCB2-1 *in vitro* and could effectively resist the oxidation of free radicals. PCB-1 could also effectively resist the D-gal induced oxidative damage effects *in vivo*. Therefore, this study supplies a scientific basis for further development and utilization of *P. cablin* polysaccharides in functional foods or medicinal industry, although the specific antioxidant mechanism needs to be further elaborated.

## Data Availability

The raw data supporting the conclusion of this article will be made available by the authors, without undue reservation.

## Abbreviations

Ara, arabinose; CAT, catalase; EDTA, ethylene diamine tetraacetie acid; Gal, galactose; GalA, galacturonic acid; Glc, glucose; GSH-Px, glutathione peroxidase; HPGPC, high-performance gel permeation chromatography; Man, mannose; MDA, malondialdehyde; •OH, hydroxyl radical; PGC-1α, peroxisome proliferator-activated receptor-gamma coactivator-1α; PVDF, polyvinylidene fluoride; Rha, rhamnose; ROS, reactive oxygen species; SIRT1, sirtuin 1; SOD, superoxide dismutase; TBA, thiobarbituric acid; TPTZ, 1,3,5- tri(2-pyridyl)-2,4,6-triazine; UCP2, uncoupling protein 2.
